# Integrative molecular subtypes of acute myeloid leukemia

**DOI:** 10.1038/s41408-023-00836-4

**Published:** 2023-05-08

**Authors:** Qianxing Mo, Seongseok Yun, David A. Sallman, Nicole D. Vincelette, Guang Peng, Ling Zhang, Jeffrey E. Lancet, Eric Padron

**Affiliations:** 1 Department of Biostatistics & Bioinformatics, H. Lee Moffitt Cancer Center & Research Institute, Tampa, FL 33612 USA; 2 Department of Malignant Hematology, H. Lee Moffitt Cancer Center & Research Institute, Tampa, FL 33612 USA; 3 Department of Clinical Cancer Prevention, The University of Texas MD Anderson Cancer Center, Houston, TX 77030 USA; 4 Department of Hematopathology and Laboratory Medicine, H. Lee Moffitt Cancer Center & Research Institute, Tampa, FL 33612 USA

**Keywords:** Cancer genomics, Acute myeloid leukaemia

**Dear Editor**,

AML is a heterogenous disease characterized by distinct clinical courses and prognoses based on genomic, epigenomic and transcriptomic profiles [1, 2]. Therefore, molecular classification and risk stratification are essential for clinical decision. Although cytogenetics is one of the most powerful prognostic indicators in AML, more than 50% of AML patients have normal karyotypes. In the past decade, advances in sequencing technology enabled incorporation of somatic mutations into molecular classification and risk stratification in AML [3, 4]. Additionally, recent studies demonstrated that gene expression profiles in leukemia stem cells and maturation state of AML cells also carry independent prognostic significance [5, 6]. Furthermore, DNA methylation patterns may provide additional prognostic values in AML [7, 8]. Although multi-omics profiles have been used to define AML molecular subtypes with distinct prognoses, they have not been systematically integrated to define integrative subtypes (iSubtypes) of AML. Therefore, there is a great clinical interest to identify AML iSubtypes and the patterns across multi-omics profiles that could be used for prognosis and targeted therapy.

In this study, we performed an integrative clustering (iCluster) [9, 10] analysis of the TCGA [1] multi-omics data including somatic mutation, DNA copy number, DNA methylation and transcriptomic data for 160 de novo adult AML samples and identified the multi-omics signatures that drove molecular classification of AML (Supplementary methods). Based on the common subtype-driver methylation and transcription signatures, we derived a 571-gene panel for classification of AML when transcriptomic data are available. Using three independent transcriptomic datasets, namely BEAT [11] (*n* = 671), GSE6891 [12] (*n* = 461) and GSE106291 [13] (*n* = 250), we demonstrated the prognostic power of the 571-gene panel in classifying AML into clinically relevant subtypes.

We identified 4 AML iSubtypes featuring distinct multi-omics signatures (Fig. 1A, B). In terms of overall survival (OS), the iSubtypes 3 was the best, the iSubtype 2 was the middle, and the iSubtypes 1 and 4 were the worst (*p* = 0.039) (Fig. 1C). At the DNA level (Fig. 1A, somatic mutation and DNA copy number), the iSubtype 1 was characterized by complex karyotypes (CK) and high-frequency mutation of *TP53* (30%) and *RUNX1* (27%); the iSubtype 2 was characterized by CK and high-frequency mutation of *CEBPA* (20%); while the iSubtypes 3 and 4 were characterized by normal karyotype (NK), deficiency of *TP53/CEBPA/RUNX1* mutations, and abundance of *FLT3/NPM1/DNMT3A* mutations, with the iSubtype 4 having higher mutation rates in the three genes than the iSubtype 3 (*FLT3*: 41% vs. 34%; *NPM1*: 57% vs. 37%; *DNMT3A*: 41% vs. 20%). At the epigenomic level (Fig. 1A, DNA methylation), the iSubtypes 1, 3, and 4 were generally characterized by hypomethylation of subtype-driver genes, while the iSubtypes 2 were generally characterized by hypermethylation of subtype-driver genes. These driver genes formed three major methylation clusters (m1-3) in which the major groups of genes were related to regulation of protein kinase activity, immune response, regulation of cell activation, leukocyte differentiation/migration and cell morphogenesis, etc. (Fig. 1D). At transcriptomic level (Fig. 1A, mRNA), the 4 iSubtypes were characterized by 3 driver gene clusters (g1-3) in which the top enriched biological processes were involved in immune process, angiogenesis, cell migration, extracellular matrix/structure organization, regulation of immune processes, etc. (Fig. 1E).

**Fig. 1:**
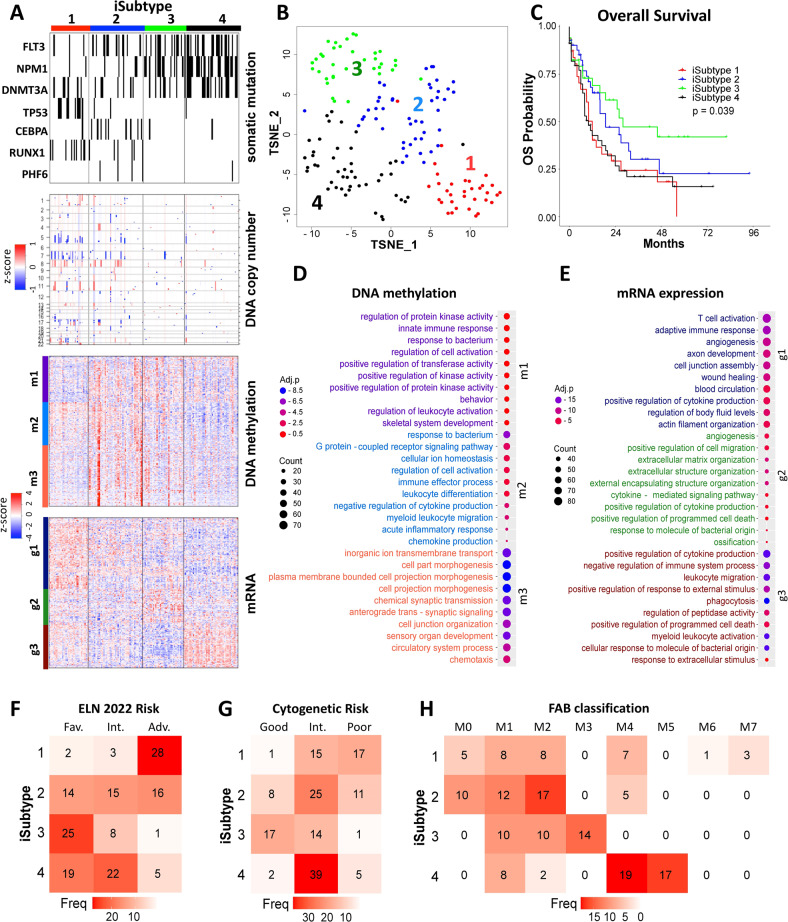
Integrative subtypes (iSubtypes) of AML. **A** Heatmaps of the characteristic multi-omics features of the 4 iSubtypes. Somatic mutation: mutation is indicated by black bar. Copy number: copy number loss, normal and gain are indicated by blue, white and red, respectively. Methylation: low, middle and high methylation are represented by blue, white and red, respectively; subtype-driver genes form three methylation clusters m1-m3 (more details in Supplementary Table 1). mRNA expression: low, middle and high expression are represented by blue, white and red, respectively; subtype-driver genes form three mRNA expression clusters g1-g3 (more details in Supplementary Table 2). **B** TCGA AML samples visualized in the 2-dimentional TSNE coordinates reduced from the 3-dimensional principal component spaces of iCluster. **C** Overall survival of the 4 iSubtypes. **D**, **E** Top 10 biological processes (GO terms) in each of the methylation clusters m1-m3 and the gene expression clusters g1-g3 (more details in Supplementary Tables 1 and 2); Adj.*p* is log10 adjusted *p*-value; Adj.*p* < –1.3 is considered statistically significant. **F**–**H** Contingency tables comparing the iSubtypes with the ELN2022 classification (**F**), the cytogenetic classification (**G**), and the FAB classification (**H**).

In comparison to the ELN2022 classification [2], 85% (28/33) of the iSubtype 1 samples were in the adverse group; 74% (25/34) of the iSubtype 3 samples were in the favorable; 89% (41/46) of the iSubtype 4 samples were in the favorable or intermediate; while the iSubtype 2 samples were almost evenly distributed in the 3 ELN2022 groups (Fig. 1F). Compared to the cytogenetic risk groups, 97% (32/33) of the iSubtype 1 samples belonged to the intermediate or poor group, 57% (25/44) of the iSubtype 2 samples belonged to the intermediate, 97% (31/32) of the iSubtype 3 samples belonged to the good or intermediate, and 85% (39/46) of the iSubtype 4 samples belonged to the intermediate (Fig. 1G). Compared to the FAB classification, the iSubtype 1 samples were distributed in various subtypes (M0, M1, M2, M4, M6 and M7); while 89% (39/44) of the iSubtype 2 samples were distributed in the M0, M1 or M2; 100% of the iSubtype 3 samples were distributed in the M1, M2 or M3; 78% (36/46) of the iSubtype 4 samples were distributed in the M4 or M5 (Fig. 1H).

There were 571 common genes identified as the subtype-drivers in the methylation and transcriptomic datasets and these genes formed three major clusters c1-3 and their overall expression patterns were negatively correlated (Fig. 2A, B). For example, the genes in cluster c3 were hypomethylated and upregulated in the iSubtype 1 and the top enriched biological processes included axon development, blood circulation, regulation of leukocyte activation and cell-cell adhesion, angiogenesis, etc. (Fig. 2C); the genes in cluster c2 were hypomethylated and upregulated in the iSubtype 4 and the top enriched biological processes included negative regulation of cytokine production, mononuclear cell differentiation, adaptive immune response, etc. (Fig. 2C). Using the mRNA expression signature of the 571 genes in the TCGA dataset as the template, the AML samples in the 3 independent transcriptomic datasets were classified into 4 transcriptomic subtypes (tSubtypes) with similar gene expression patterns (Fig. 2D–F). The OS of the 4 subtypes in these three cohorts also had similar trends in which the subtypes 2 and 3 had a better OS, compared to the subtypes 1 and 4 (Fig. 2G–I).

**Fig. 2:**
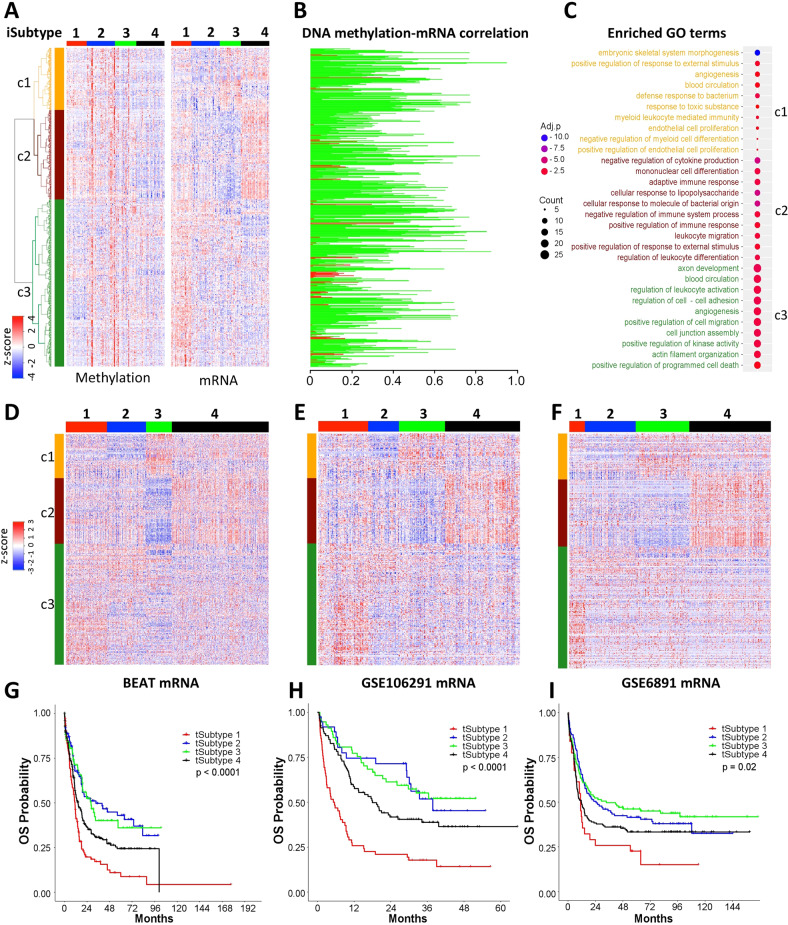
Prognostic power of the iSubtype-driver gene expression signatures. **A** Heatmaps of 571 common genes in the TCGA methylation and gene expression datasets that form 3 major gene clusters c1-c3 (more details in Supplementary Table 3). **B** Pearson correlation coefficients of gene methylation and expression (genes are arranged in the same order as those on Fig. 2A). Negative and positive correlations are represented by green and red, respectively. **C** Top 10 most enriched biological processes (GO terms) in each of the gene clusters c1-c3 (more details in Supplementary Table 3); Adj.*p* is log10 adjusted *p*-value; Adj.*p* < –1.3 is considered statistically significant. **D**–**F** Heatmaps of gene expression in the BEAT, GSE106291 and GSE6891 cohorts. The samples were classified using the mRNA signature of Fig. 2A and 5-nearest neighbor method. **G**–**I** Overall survival of the 4 transcriptomic subtypes (tSubtypes) in the BEAT, GSE106291, and GSE6891 cohorts.

In summary, iCluster analysis generated an integrative molecular portrait of AML and revealed the correlations among multi-omics profiles that determined the molecular classification of AML, which was not revealed previously by individual-omics data analysis. For example, based on the copy number data, AML could be divided into CK-AML (iSubtype 1 + 2) and NK-AML (iSubtype 3 + 4). However, the OS was not significantly different between iSubtype 1 + 2 and iSubtype 3 + 4 (*p* = 0.7), implying that using copy number data alone is not sufficient to stratify AML into clinically meaningful subgroups. By integrating the other omics data, iCluster further divided the CK-AML into iSubtypes 1 and 2, and the NK-AML into iSubtypes 3 and 4, respectively. The iSubtype 1 had an inferior OS than the iSubtype 2, which might be due to its much higher mutation rate of *TP53* (30% in 1 vs. 4% in 2). These observations are consistent with the reports that a subgroup of CK-AML/MDS (myelodysplastic syndromes) with mutated *TP53* (*mTP53*) had a worse prognosis than the subgroup with CK alone [14, 15]. Gene set enrichment analysis of the Hallmark pathways showed that a wide range of pathways were significantly elevated in the iSubtupe 1, compared to the iSubtype 2 (Supplementary Fig. 1A). These elevated pathways were mainly involved in cancer development (e.g., epithelial mesenchymal transition, angiogenesis), DNA damage response (e.g., UV response downregulated genes), immune response (e.g., complement, coagulation, inflammatory response, allograft rejection), and signaling (e.g., *WNT* beta catenin, *TGF* beta, *KRAS*, *NOTCH*, hedgehog, *IL2*_*STAT5*), and cellular component organization (e.g., apical junction, apical surface). Additionally, *CTLA4* and *PDL1* had a significantly higher expression in the iSubtype 1 compared to the other iSubtypes, which could be potential targets for anti-*CTLA4* and anti-*PDL1* therapies (Supplementary Fig. 2).

It is still a challenge to classify NK-AML into subgroups for prognosis and target therapy. By iCluster analysis, the NK-AML iSubtypes 3 and 4 were distinguished by *FLT3/NPM1/DNMT3A* mutation, methylation and gene expression levels. Compared to the iSubtype 3, the most significantly elevated Hallmark pathways in the iSubtype 4 were mainly involved in immune response (e.g., interferon gamma/alpha response, inflammatory response, complement, allograft rejection, *IL6_JAK_STAT3* signaling, coagulation), signaling (e.g., *TNFA* signaling via *NFkB*, *KRAS*, *IL2_STAT5*), proliferation (e.g., *P53* pathway) and metabolism (e.g., xenobiotics metabolism) (Supplementary Fig. 1B). Furthermore, a cluster of genes involved in negative regulation immune system process (e.g., negative regulation of T cell proliferation: *CD86*, *GPNMB, CEBPB, CLEC4G*, *VSIG4*); negative regulation of T cell receptor signaling: *LGALS3*, *PTPRJ, LAPTM5*) had an elevated expression in the iSubtype 4 (Supplementary Fig. 3), which are potential targets for immunotherapy. When only transcriptomic data were available, we demonstrated that the 571-gene panel derived from the driver methylation and transcriptomic signatures had an excellent prognostic power in classifying AML into transcriptomic subtypes with similar OS in the 3 independent cohorts. Notably, the expression patterns of the genes involved in negative regulation of immune system process in the TCGA mRNA data were confirmed in the 3 independent cohorts (Supplementary Fig. 3).

Currently, diagnosis and management of AML are heavily dependent on genetics-based risk classification such as ELN2022. Overall, the risk classifications of AML by iCluster, ELN2022 and cytogenetics were comparable (Supplementary Fig. 4). Remarkably, 88% of the adverse group of ELN2022 and 82% of the poor group of cytogenetics were CK-AML (iSubtype 1 or 2), indicating a high concordance of classification for CK-AML by iCluster and genetics-based approaches. By integrating methylation and gene expression data that were not routinely incorporated in clinical practice, we demonstrated that they were useful in classifying AML into clinically meaningful groups. For example, although 89% of the NK-AML iSubtype 4 samples belonged to the favorable or intermediate group of ELN2022 and cytogenetics, it had an OS as poor as the CK-AML iSubtype 1. The gene expression signatures may be further explored for prognosis and target therapy for NK-AML.

## Supplementary information

Supplementary Materials

Supplementary Table 1

Supplementary Table 2

Supplementary Table 3

## Data Availability

The AML multi-omics data were available at http://firebrowse.org/; the other 3 transcriptomic data were available at https://biodev.github.io/BeatAML2/ and https://www.ncbi.nlm.nih.gov/geo/ under access numbers GSE106291 and GSE6891.

## References

[1] Ley TJ, Miller C, Ding L, Raphael BJ, Mungall AJ, Cancer Genome Atlas Research N (2013). Genomic and epigenomic landscapes of adult de novo acute myeloid leukemia. N Engl J Med.

[2] Döhner H, Wei AH, Appelbaum FR, Craddock C, DiNardo CD, Dombret H (2022). Diagnosis and management of AML in adults: 2022 recommendations from an international expert panel on behalf of the ELN. Blood.

[3] Papaemmanuil E, Gerstung M, Bullinger L, Gaidzik VI, Paschka P, Roberts ND (2016). Genomic classification and prognosis in acute myeloid leukemia. New Engl J Med.

[4] Tyner JW, Tognon CE, Bottomly D, Wilmot B, Kurtz SE, Savage SL (2018). Functional genomic landscape of acute myeloid leukaemia. Nature.

[5] Elsayed AH, Rafiee R, Cao X, Raimondi S, Downing JR, Ribeiro R (2020). A six-gene leukemic stem cell score identifies high risk pediatric acute myeloid leukemia. Leukemia.

[6] Ng SWK, Mitchell A, Kennedy JA, Chen WC, McLeod J, Ibrahimova N (2016). A 17-gene stemness score for rapid determination of risk in acute leukaemia. Nature.

[7] Deneberg S, Guardiola P, Lennartsson A, Qu Y, Gaidzik V, Blanchet O (2011). Prognostic DNA methylation patterns in cytogenetically normal acute myeloid leukemia are predefined by stem cell chromatin marks. Blood.

[8] Figueroa ME, Lugthart S, Li Y, Erpelinck-Verschueren C, Deng X, Christos PJ (2010). DNA methylation signatures identify biologically distinct subtypes in acute myeloid leukemia. Cancer Cell.

[9] Mo Q, Shen R, Guo C, Vannucci M, Chan KS, Hilsenbeck SG (2018). A fully Bayesian latent variable model for integrative clustering analysis of multi-type omics data. Biostatistics.

[10] Mo Q, Wang S, Seshan VE, Olshen AB, Schultz N, Sander C (2013). Pattern discovery and cancer gene identification in integrated cancer genomic data. Proc Natl Acad Sci USA.

[11] Bottomly D, Long N, Schultz AR, Kurtz SE, Tognon CE, Johnson K (2022). Integrative analysis of drug response and clinical outcome in acute myeloid leukemia. Cancer Cell.

[12] de Jonge HJ, Valk PJ, Veeger NJ, ter Elst A, den Boer ML, Cloos J (2010). High VEGFC expression is associated with unique gene expression profiles and predicts adverse prognosis in pediatric and adult acute myeloid leukemia. Blood.

[13] Herold T, Jurinovic V, Batcha AMN, Bamopoulos SA, Rothenberg-Thurley M, Ksienzyk B (2018). A 29-gene and cytogenetic score for the prediction of resistance to induction treatment in acute myeloid leukemia. Haematologica.

[14] Haase D, Stevenson KE, Neuberg D, Maciejewski JP, Nazha A, Sekeres MA (2019). TP53 mutation status divides myelodysplastic syndromes with complex karyotypes into distinct prognostic subgroups. Leukemia.

[15] Weinberg OK, Siddon A, Madanat YF, Gagan J, Arber DA, Dal Cin P (2022). TP53 mutation defines a unique subgroup within complex karyotype de novo and therapy-related MDS/AML. Blood Adv.

