# Supplementary material for: Integrative molecular subtypes of acute myeloid leukemia
Source: Blood Cancer J. 2023 May 8;13(1):71. doi: 10.1038/s41408-023-00836-4 (PMC10167212; doi:10.1038/s41408-023-00836-4)
Supplement: Supplementary file 1 — Supplementary Materials [file 41408_2023_836_MOESM1_ESM.pdf]

Supplementary Methods and Figures

Integrative Molecular Subtypes of Acute Myeloid Leukemia

Qianxing Mo[1,*,#], Seongseok Yun[2,*], David Sallman[2], Nicole Vincelette[2], Guang Peng[3], Ling Zhang[4], Jeffrey Lancet[2], Eric Padron[2]

1. Department of Biostatistics & Bioinformatics, H. Lee Moffitt Cancer Center & Research Institute, Tampa, FL 33612, USA
2. Department of Malignant Hematology, H. Lee Moffitt Cancer Center & Research Institute, Tampa, FL 33612, USA
3. Department of Clinical Cancer Prevention, The University of Texas MD Anderson Cancer Center, Houston, TX 77030, USA
4. Department of Hematopathology and Laboratory Medicine, H. Lee Moffitt Cancer Center & Research Institute, Tampa, FL 33612, USA

[*]Q. M. and S. Y. are co-first authors.

[#]Correspondence: Qianxing Mo, Department of Biostatistics & Bioinformatics, H. Lee Moffitt Cancer Center & Research Institute, 12902 Magnolia Drive, Tampa, FL 33612, USA.

email: qianxing.mo@moffitt.org

ORCID profiles: Q.M., 0000-0002-0021-7694;

Supplementary Methods

Integrative clustering (iCluster) analysis

All the multi-omics data used for this study were publicly available. As part of previously published studies, the informed consent from each subject for collection and molecular profiling analysis of the tumor samples was approved by individual Institutional Review Board (IRB). The AML multi-omics data were generated by TCGA[1] and the level 3 data were publicly available at http://firebrowse.org/. There were 160 *de novo* adult AML samples with multi-omics data including somatic mutation, DNA copy number, DNA methylation and RNA-seq transcriptomic data, which were used for iCluster analysis. The multi-omics data were processed to form 4 data matrices (e.g., matrices with *n* samples and *m* features) for iCluster analysis as described previously [2, 3]. For the somatic mutation data, a gene mutation status was coded as 1 if the gene had any of the mutations including in-frame deletion/insertion, frame shift deletion/insertion, missense/nonsense/nonstop mutation, RNA, splice site and translation start site; otherwise the gene's mutation status was coded as 0. Genes with mutation rate ≥ 2% in the samples were selected for iCluster analysis. The copy number data were log2 ratios of tumor/normal chromosomal segment means in which the adjacent segment means were merged using the function *CNregions* in the Bioconductor R package iClusterPlus [4] (https://bioconductor.org/packages/release/bioc/html/iClusterPlus.html). Only the merged regions on autosomes were used for iCluster analysis. The methylation data were logit-transformed beta values and the top 25% most variable genes were used for iCluster analysis. The mRNA expression data were log2-transformed values of the RNA-seq normalized counts plus 1 and the top 25% most variable genes were used for iCluster analysis. The iCluster analysis of the 4 omics data was performed using the iClusterBayes method [5]. To find an optimal number of clusters, we tested the cluster number parameter K from 1 to 6 and found the 4-clusters (K=3) solution was optimal. In the somatic mutation, DNA copy number and methylation datasets, genes with posterior probability of >0.9 were shown on Figure 1A, which were considered to be the major drivers of integrative clustering. For the DNA copy number data, all the merged autosome regions were shown on Figure 1A.

Bioinformatic analysis

To investigate the prognostic value of the subtype-driver genes, we performed classification analysis of AML samples using the TCGA mRNA expression data as the training dataset and the other three mRNA expression datasets, namely BEAT [6] (n = 671), GSE6891 [7] (n = 461), GSE106291 [8] (n = 250) as the testing datasets. The normalized mRNA expression for all the datasets were used for classification analysis. The BEAT data were publicly available at https://biodev.github.io/BeatAML2/. The other two mRNA expression datasets were publicly available at the GEO database (https://www.ncbi.nlm.nih.gov/geo/) with accession numbers GSE6891 and GSE106291. For the microarray mRNA expression dataset GSE6891, the standard deviations of the expression values of the untreated patients were much larger than those of the treated patients. Therefore, we only used the 461 samples of the treated patients for the classification analysis. In addition, if a gene's expression was measured by multiple probes, we selected the probe with the largest variance to represent the gene. To perform classification

analysis, the gene expression values for each sample were standardized by subtracting the sample mean and then dividing the sample standard deviation. The k-nearest neighbor method (*knn* function in the R package class) was used for the classification analysis, where k was set to 5. The enrichment analysis of gene ontology (GO) terms of biological process was performed using the *enrichGO* function of the R package clusterProfiler [9] and all the genes in the data were used as the background genes. The redundant GO terms were removed using the *simplify* function with similarity cutoff of 0.5. To correct for multiple comparisons, the raw p-values were adjusted using Benjamini-Hochberg method. All the bioinformatic analyses were perform using R version 4.1.2 (https://cran.r-project.org/). Gene set enrichment analysis of the Hallmark pathways in the Molecular Signature Database (MSigDB) [10] was performed using software GSEA 3.0 (https://www.gsea-msigdb.org/gsea).

Statistical analysis

Overall survival was estimated by Kaplan–Meier method. Log-rank test was used to compare overall survival of the patient groups. Kruskal-Wallis test was used to compare CTLA4 and PD-L1 expression among the 4 iSubtypes. All the statistical analyses were performed using R 4.1.2 (https://www.r-project.org). P values were two-sided and p-value < 0.05 was considered statistically significant.

Classification of TCGA AML samples

Based on the European LeukemiaNet 2022 (ELN 2022) guidance [11], we classified the TCGA AML samples into favorable, intermediate, and adverse risk groups. The cytogenetic risk classification (good, intermediate, and poor) and FAB classification (M0-7) for the TCGA AML samples were obtained from the published data by Zeng et al. [12]

Code availability

The *iClusterBayes* function in the R package *iClusterPlus* (v. 1.22.0) (https://bioconductor.org/packages/release/bioc/html/iClusterPlus.html) was used for the iCluster analysis of the TCGA AML multi-omics data. R code used for the analyses can be obtained from the corresponding author upon request.

Supplementary Figures

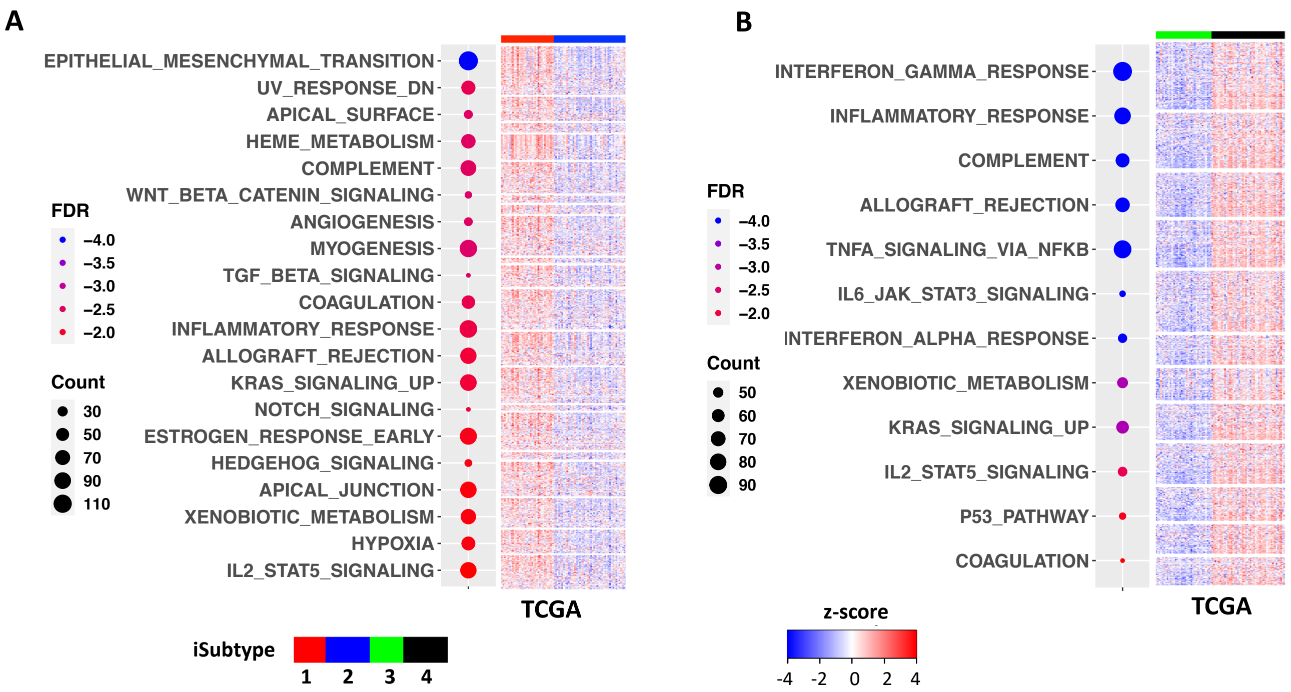

Figure 1. Up-regulated Hallmark pathways in the TCGA mRNA data. **A**: Up-regulated pathways in iSubtype 1, compared to iSubtype 2. **B**: Up-regulated pathways in iSubtype 4, compared to iSubtype 3.

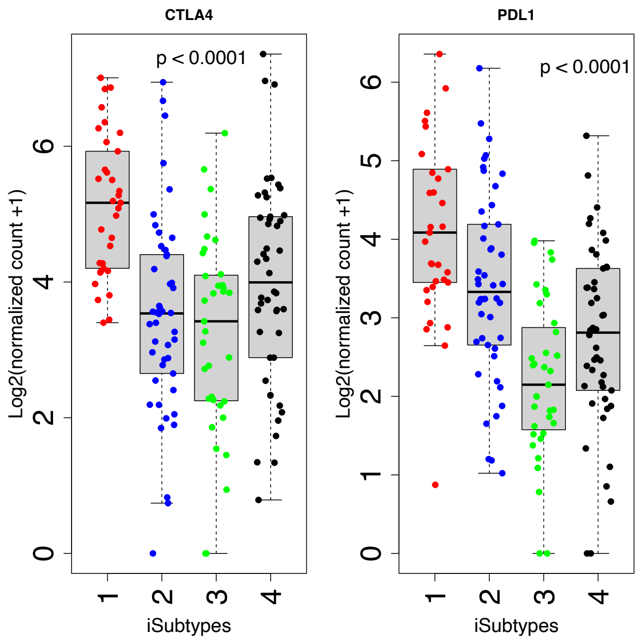

Figure 2. Expression of *CTLA4* and *PDL1* in the iSubtypes of AML.

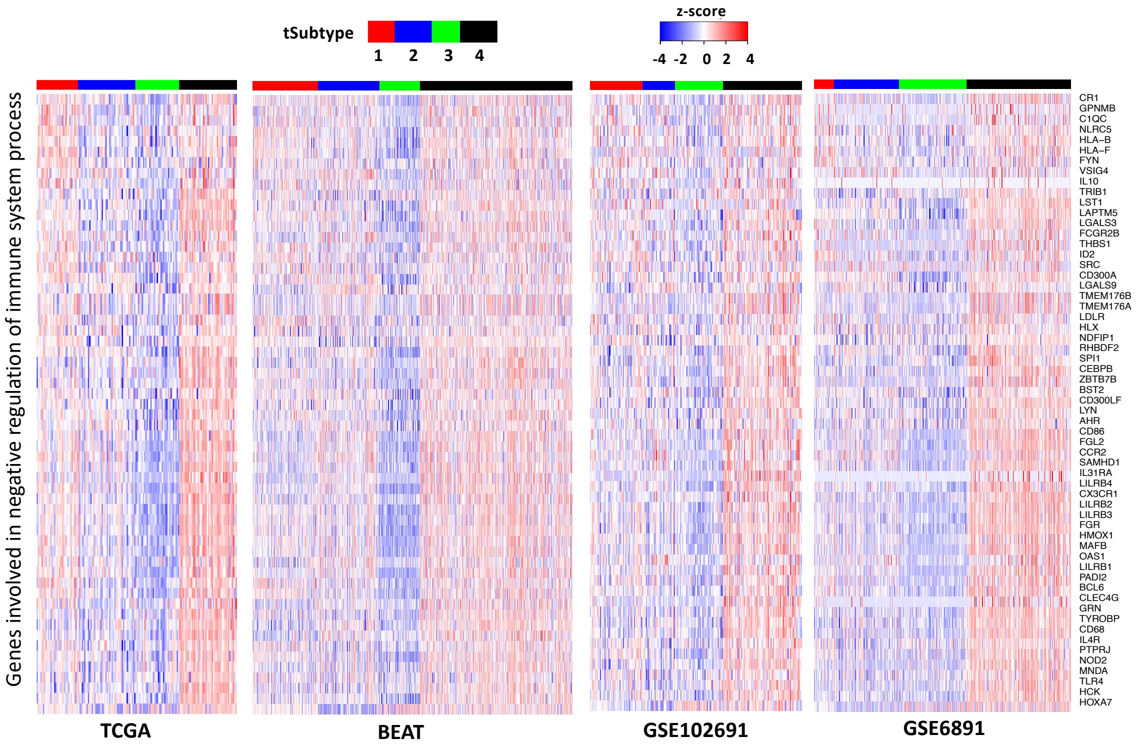

Figure 3. Expression pattern of genes involved in negative regulation of immune system process in the TCGA, BEAT, GSE102691 and GSE6891 mRNA datasets.

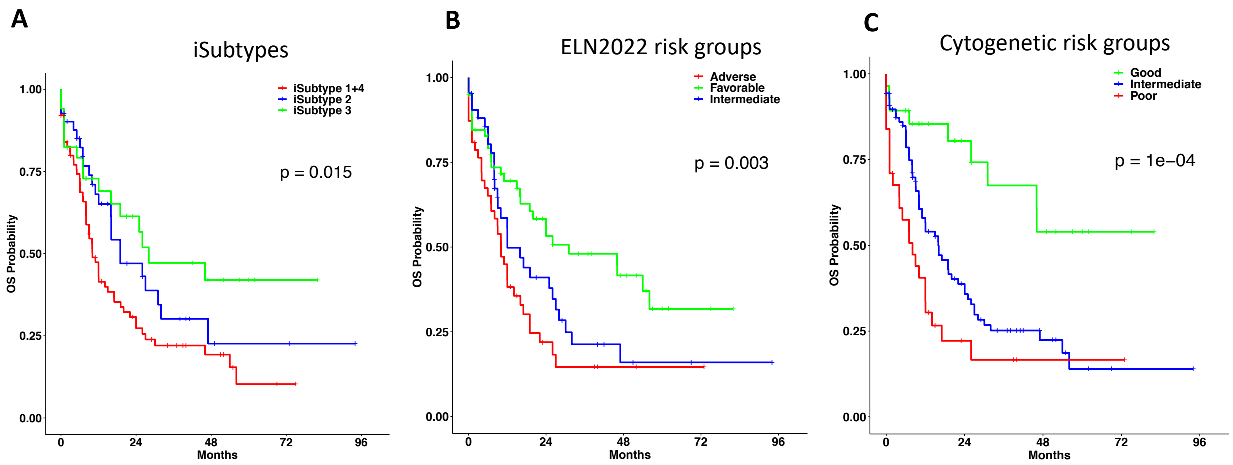

Figure 4. Overall survival (OS) of iSubtypes (**A**), ELN2022 (**B**) and cytogenetic risk (**C**) groups. The concordance probability estimates (CPE)[13] of the iSubtypes, ELN 2022 and Cytogenetic risk groups are 0.58, 0.59 and 0.62, respectively. Since the iSubtypes 1 and 4 have similar OS, they are combined to form the iSubtype 1+4 (shown on **A**). The CPE for the 4 uncombined iSubtypes is also 0.58.

Reference

1. Cancer Genome Atlas Research N, Ley TJ, Miller C, Ding L, Raphael BJ, Mungall AJ*, et al.* Genomic and epigenomic landscapes of adult de novo acute myeloid leukemia. *N Engl J Med* 2013 May 30; **368**(22)**:** 2059-2074.

2. Mo Q, Li R, Adeegbe DO, Peng G, Chan KS. Integrative multi-omics analysis of muscle-invasive bladder cancer identifies prognostic biomarkers for frontline chemotherapy and immunotherapy. *Commun Biol* 2020 Dec 17; **3**(1)**:** 784.

3. Mo Q, Wan L, Schell MJ, Jim H, Tworoger SS, Peng G. Integrative Analysis Identifies Multi-Omics Signatures That Drive Molecular Classification of Uveal Melanoma. *Cancers (Basel)* 2021 Dec 7; **13**(24).

4. Mo Q, Wang S, Seshan VE, Olshen AB, Schultz N, Sander C*, et al.* Pattern discovery and cancer gene identification in integrated cancer genomic data. *Proc Natl Acad Sci U S A* 2013 Mar 12; **110**(11)**:** 4245-4250.

5. Mo Q, Shen R, Guo C, Vannucci M, Chan KS, Hilsenbeck SG. A fully Bayesian latent variable model for integrative clustering analysis of multi-type omics data. *Biostatistics* 2018 Jan 1; **19**(1)**:** 71-86.

6. Bottomly D, Long N, Schultz AR, Kurtz SE, Tognon CE, Johnson K*, et al.* Integrative analysis of drug response and clinical outcome in acute myeloid leukemia. *Cancer Cell* 2022; **40**(8)**:** 850-864.e859.

7. de Jonge HJ, Valk PJ, Veeger NJ, ter Elst A, den Boer ML, Cloos J*, et al.* High VEGFC expression is associated with unique gene expression profiles and predicts adverse prognosis in pediatric and adult acute myeloid leukemia. *Blood* 2010 Sep 9; **116**(10)**:** 1747-1754.

8. Herold T, Jurinovic V, Batcha AMN, Bamopoulos SA, Rothenberg-Thurley M, Ksienzyk B*, et al.* A 29-gene and cytogenetic score for the prediction of resistance to induction treatment in acute myeloid leukemia. *Haematologica* 2018 Mar; **103**(3)**:** 456-465.

9. Yu G, Wang LG, Han Y, He QY. clusterProfiler: an R package for comparing biological themes among gene clusters. *OMICS* 2012 May; **16**(5)**:** 284-287.

10. Liberzon A, Birger C, Thorvaldsdottir H, Ghandi M, Mesirov JP, Tamayo P. The Molecular Signatures Database (MSigDB) hallmark gene set collection. *Cell Syst* 2015 Dec 23; **1**(6)**:** 417-425.

11. Döhner H, Wei AH, Appelbaum FR, Craddock C, DiNardo CD, Dombret H*, et al.* Diagnosis and management of AML in adults: 2022 recommendations from an international expert panel on behalf of the ELN. *Blood* 2022; **140**(12)**:** 1345-1377.

12. Zeng AGX, Bansal S, Jin L, Mitchell A, Chen WC, Abbas HA*, et al.* A cellular hierarchy framework for understanding heterogeneity and predicting drug response in acute myeloid leukemia. *Nat Med* 2022 Jun; **28**(6)**:** 1212-1223.

13. Heller G, Mo Q. Estimating the concordance probability in a survival analysis with a discrete number of risk groups. *Lifetime Data Anal* 2016 Apr; **22**(2)**:** 263-279.
